# Prolonged Prophylactic Ureteral Stent Placement and BK Polyomavirus Infection After Renal Transplantation—A Retrospective Case-control Study

**DOI:** 10.1016/j.euros.2025.10.014

**Published:** 2025-11-04

**Authors:** Haris Omić, Simon Hoffmann, Michael Eder, Robert Strassl, Daniela Gerges, Shahrokh F. Shariat, Željko Kikić

**Affiliations:** a Division of Nephrology and Dialysis, Department of Medicine III, Medical University of Vienna, Vienna, Austria; b Department of Virology, Medical University of Vienna, Vienna, Austria; c Department of Urology, Medical University of Vienna, Vienna, Austria; d Hourani Center for Applied Scientific Research, Al-Ahliyya Amman University, Amman, Jordan; e Weill Medical College of Cornell University, New York, NY, USA; f Department of Urology, University of Texas Southwestern, Dallas, TX, USA; g Karl Landsteiner Institute of Urology and Andrology, Vienna, Austria; h Centre for Translational Medicine, Semmelweis University, Budapest, Hungary

**Keywords:** BK polyomavirus, Kidney transplantation, Ureteral stent, BK polyomavirus DNAemia, Stent duration

## Abstract

**Background and objective:**

BK polyomavirus (BKPyV) poses a significant challenge in kidney transplant (KTX) recipients, potentially leading to BKPyV-associated nephropathy. Prophylactic ureteral stent (UrSt) placement is the standard care following KTX. Emerging data suggest that UrSt may influence the incidence and severity of BKPyV-DNAemia. We hypothesized a dose-dependent relationship between an indwelling UrSt and the risk of BKPyV-DNAemia in KTX recipients.

**Methods:**

We included all KTX recipients with detectable BKPyV-DNAemia at the Medical University of Vienna from 2010 to 2021; those without DNAemia served as controls. This nested, retrospective, 1:1 sex- and age-matched case-control study assessed whether UrSt placement and/or its duration was associated with BKPyV.

**Key findings and limitations:**

Of 438 KTX recipients, 51.6% of viremic patients had received UrSt, compared with 47% of nonviremic controls. While UrSt placement was not associated with the risk of BKPyV-DNAemia (odds ratio [OR] 1.20, 95% confidence interval [CI] 0.83–1.75), indwelling time of >8 wk was (OR 1.79, 95% CI 1.10–2.93, *p* = 0.02). In a multivariable analysis, it remained an independent predictor of BKPyV-DNAemia (adjusted OR 1.74, 95% CI 1.06–2.86, *p* = 0.03). Limitations include the retrospective, single-center study design.

**Conclusions and clinical implications:**

Prophylactic UrSt placement after KTX does not increase the risk of developing BKPyV-DNAemia. However, the indwelling time of the UrSt should be limited to <8 wk to reduce the risk of BKPyV-DNAemia. Therefore, early removal of UrSt—preferably within 8 wk—should be pursued when clinically feasible. Further research is needed to define the optimal UrSt indwelling time and clarify the underlying pathophysiological mechanisms.

**Patient summary:**

In this study, we examined whether the placement and duration of ureteral stents after kidney transplantation affect the risk of developing a BK polyomavirus that can harm the transplanted organ. We found that the length of time the stent remains in place—especially beyond 8 weeks—was linked to a higher risk of viral reactivation. Removal of the stent earlier may help protect patients from these complications.

## Take Home Message

Prolonged ureteral stent indwelling times beyond 8 wk—not stent placement itself—is associated with an increased risk of BK polyomavirus reactivation after kidney transplantation.

## 1 Introduction

BK polyomavirus (BKPyV)-associated nephropathy (BKPyVAN) remains a leading cause of graft dysfunction and graft loss in kidney transplant (KTX) recipients, affecting up to 10% of the patients, and, to date, there is no effective therapy [[1], [2], [3], [4], [5], [6], [7], [8], [9], [10]]. Childhood exposure leaves BKPyV latent in renal tubular and urothelial cells, and standard immunosuppression (IS)—induction with IL-2 receptor antagonists or lymphocyte-depleting agents, followed by tacrolimus, mycophenolate mofetil (MMF), and glucocorticoids—weakens the host antiviral defenses, leading to viral reactivation. Disease severity ranges from asymptomatic BKPyV-DNAemia to biopsy-proven BKPyVAN, causing early graft loss in up to 30% of BKPyVAN patients [11,12].

Additionally, BKPyV reactivation can trigger hemorrhagic cystitis, particularly in hematopoietic stem cell transplant recipients, with the incidence ranging from 7% to 54% and severity reaching painful hematuria resulting in extended hospital stays [13]. Moreover, growing evidence implicates BKPyV in the development of urothelial carcinomas after transplant, with case series describing BKPyV-positive bladder cancers in KTX and bone marrow transplant recipients [14].

Contrary to the established drivers of BKPyV replication such as tacrolimus-based regimens, delayed graft function (DGF), and recipient age, the role of ureteral stent (UrSt) placement remains less clear [[15], [16], [17], [18], [19], [20]]. Universally deployed to prevent ureteroneocystostomy leaks and obstruction after KTX, a prophylactic UrSt has been suggested to induce BKPyV replication via a “two-hit” mechanism: mechanical epithelial trauma during insertion, followed by IS-driven viral proliferation [[21], [22], [23], [24], [25], [26], [27], [28], [29]]. Retrospective studies report higher BKPyV-DNAemia and BKPyVAN rates in stented versus unstented patients; however, definitive conclusions are lacking due to small sample sizes and heterogeneity of the investigated cohorts [[28], [29], [30]]. While prophylactic UrSt placement itself may contribute to BKPyV reactivation, the impact of the duration of UrSt indwelling time remains poorly investigated. As the 2017 update of the European Association of Urology (EAU) guidelines strongly recommended prophylactic UrSt placement in KTX recipients, and given the limitations of prior studies, we consecutively conducted a nested, retrospective, 1:1 sex- and age-matched analysis of 438 KTX recipients [31]. Our hypothesis addressed (1) whether prophylactic UrSt placement per se increases the incidence of BKPyV-DNAemia and BKPyVAN, and (2) whether the duration of UrSt indwelling time impacts the risk of BKPyVAN—in a sex- and age-matched cohort.

## 2 Patients and methods

### 2.1 Study population

After the approval of the Ethics Committee of the Medical University of Vienna (EC-Nr 1776/2022), all KTX recipients (≥18 yr old) at the Vienna General Hospital were screened from 2010 to 2021 for study inclusion. The study was reported following the STROBE guidelines. Screening for BKPyV-DNAemia at our center was conducted following international guidelines, using quantitative polymerase chain reaction (qPCR) to monitor BKPyV-DNAemia. BKPyV-DNAemia was interpreted as viral reactivation and was detected by qPCR targeting conserved viral regions, which reliably identify all major genotypes without distinction. Genotyping was not performed. Patients with BKPyV-DNAemia (>10^2^ copies per milliliter [c/ml]) were assigned to the case group, and patients without BKPyV-DNAemia were assigned to the control group. Data on donor, transplant, and recipient characteristics were obtained retrospectively. Cases and controls were matched in a 1:1 ratio according to sex and age at KTX. Prophylactic ureteral stenting was introduced at our center in 2017, following the EAU guidelines [31]. Before this period, prophylactic UrSt placement was based on the physician’s preference. At our center, UrSts are removed routinely at 8 wk after transplant. In cases where indwelling time exceeded 8 wk, this was typically due to minor delays or clinical issues requiring deviation from standard practice, rather than predefined medical indications. For all patients, implantation, explantation, and precise indwelling time were documented systematically.

### 2.2 Primary and secondary endpoints

Detection of any BKPyV-DNAemia (>10^2^ c/ml) within 12 mo after KTX was defined as the primary endpoint. The secondary endpoints included peak BKPyV-DNAemia, time to BKPyV-DNAemia, occurrence of BKPyVAN, and frequency of graft loss. For a secondary analysis, UrSt indwelling time was categorized as short (<4 wk), intermediate (4–8 wk), and long (>8 wk) according to the present heterogeneous approaches [[32], [33], [34]]. Similarly, levels of BKPyV-DNAemia were defined as low (<10^3^ c/ml), intermediate (10^3^–10^4^ c/ml), or high (>10^4^ c/ml) [10]. A subgroup analysis focusing on the period before ureteral stenting became the standard practice (before and after 2017) was assessed to evaluate whether the impact of UrSt use on BKPyV reactivation varied across these distinct timeframes.

### 2.3 Statistical analysis

Continuous variables were assessed for normality using the Shapiro-Wilk test. Normally distributed data were presented as mean ± standard deviation, and non‐normally distributed data were presented as median and interquartile range. Categorical variables were summarized as counts and percentages. Baseline characteristics of cases (BKPyV-DNAemia) and controls were compared to assess the differences regarding other proposed risk factors. UrSt placement as a risk factor was compared between the two groups using a χ^2^ test. UrSt indwelling time and BKPyV-DNAemia were categorized as mentioned above (short [<4 wk], intermediate [4–8 wk], and long [>8 wk]). The Kruskal-Wallis test was performed to test for statistical differences between the categories of these variables. A binary logistic regression model was used to identify the independent predictors of BKPyV-DNAemia and BKPyVAN. Risk factors that were statistically significant in a univariable analysis were included in a subsequent multivariable analysis. Spearman correlations were calculated between BKPyV-DNAemia and BKPyV-DNAuria. An additional multivariable logistic regression was conducted to assess whether the association between UrSt implantation/indwelling time and BKPyV‐DNAemia/BKPyVAN differed by transplant era. Interaction significance was evaluated by Wald χ^2^ tests on the product term; a *p* value of <0.05 was considered statistically significant throughout the study. A statistical analysis was performed using the commercially available IBM SPSS Statistics software (version 29.0.2.0.for Mac) and GraphPad Prism (GraphPad Prism 10.0.3 [217] Macintosh version, 1994–2023; Software MacKiev, LLC).

### 2.4 Sample size calculation

An a priori sample size calculation was performed for a 1:1 matched case-control study investigating the association between UrSt placement and BKPyV-DNAemia. Previous studies have reported odds ratios (ORs) for this association ranging from approximately 1.55 to 4.71 [24,25,29]. Based on these findings and the need for a balanced yet clinically meaningful effect size, we selected an OR of 2.0 as our target. An exposure prevalence of BKPyV-DNAemia of 30% among the study population was assumed from relevant literature [25,27,29,35,36].

Using standard formulas for matched case-control designs, a two-sided alpha of 0.05, and 80% power, we determined that 219 matched pairs (219 cases and 219 controls) would be required to detect an OR of 2.0 for the association between UrSt placement and BKPyV-DNAemia. To account for an anticipated 10% exclusion rate due to missing data or loss to follow-up, we identified 235 eligible cases. After excluding 16 cases due to incomplete data, the final analytic cohort comprised exactly 219 matched pairs, thus meeting the calculated sample size requirements for adequate statistical power.

## 3 Results

### 3.1 Baseline characteristics

In total, 235 KTX recipients tested positive for BKPyV-DNAemia within 12 mo after KTX. Sixteen patients who were lost to follow-up were excluded. The final cohort consisted of 219 cases and 219 controls, in total 438 patients (Supplementary Fig. 1). Matching was based on sex and age at KTX in a 1:1 ratio. Both groups were comparable regarding the relevant risk factors for BKPyV-DNAemia, including sex, age at KTX, renal replacement therapy before KTX, cytomegalovirus risk, human leukocyte antigen mismatch, peak panel reactive antibodies, donor age, and DGF (Table 1). Maintenance IS consisted of tacrolimus, MMF, and glucocorticoids in the majority of patients. Patients with detectable BKPyV-DNAemia were more likely to receive tacrolimus-based IS regimens (*p* < 0.01) and glucocorticoids (*p* = 0.02).

**Table 1:** Demographic and KTX-related parameters of cases and controls

Variable	BKPyV-DNAemia (*n* = 219)	No BKPyV-DNAemia (*n* = 219)	*p* value
*Demographic data*			
Male sex, *n* (%)	150 (68.5)	150 (68.5)	>0.99
Age at KTX, median (IQR)	57 (47–66)	57 (47–65)	0.87
First KTX, *n* (%)	182 (83.1)	168 (76.7)	0.09
Prophylactic UrSt implantation, *n* (%)	113 (51.6)	103 (47.0)	0.34
UrSt indwelling time (d), median (IQR)	62 (42–93)	47 (39–66)	<0.01
*Underlying disease*			
Diabetic nephropathy, *n* (%)	26 (11.9)	33 (15.1)	0.33
Glomerulonephritis, *n* (%)	41 (18.7)	50 (22.8)	0.29
Polycystic kidney disease, *n* (%)	36 (16.4)	28 (12.8)	0.28
Malignancies, *n* (%)	5 (2.3)	6 (2.7)	0.76
Vascular origin, *n* (%)	16 (7.3)	22 (10)	0.31
Other a, *n* (%)	76 (34.7)	65 (29.7)	0.26
Unknown, *n* (%)	19 (8.7)	15 (6.8)	0.47
*KTX-related data*			
RRT prior to KTX, *n* (%)	193 (88.1)	201 (91.8)	0.20
CMV high risk, *n* (%)	46 (21.0)	42 (19.2)	0.63
HLA mismatch, median (IQR)	3 (2–4)	3 (2–4)	0.42
Peak PRA, mean (SD)	8 (21)	8 (±20)	0.1
Donor age, median (IQR)	54 (43–68)	55 (44–65)	0.98
*Immunosuppression*			
Tacrolimus, *n* (%)	218 (99.5)	209 (95.4)	<0.01
MMF, *n* (%)	216 (98.6)	212 (96.8)	0.20
Glucocorticoids, *n* (%)	219 (100)	214 (97.7)	0.02
*Induction therapy*	24 (11.0)	26 (11.9)	0.76
Basiliximab, *n* (%)	56 (25.6)	83 (37.9)	<0.01
IAS + plasmapheresis, *n* (%)	20 (9.1)	22 (10.0)	0.75
*KTX and follow-up*			
DGF, *n* (%)	51 (23.3)	64 (29.2)	0.16
eGFR 1 yr after KTX (ml/min/1.73 m*^2^*), mean (SD)	41.09 (±13.08)	40.84 (±13.68)	0.89
P/C-R 1 yr after KTX (mg/g), median (IQR)	153 (87–300.5)	126 (81–268)	0.50
Graft loss, *n* (%)	4 (1.8)	3 (1.4)	0.70

BKPyV = BK polyomavirus; CMV = cytomegalovirus; DGF = delayed graft function; eGFR = estimated glomerular filtration rate; HLA = human leukocyte antigen; IAS = immunoadsorption; IQR = interquartile range; KTX = kidney transplantation; MMF = mycophenolate mofetil; P/C-R = protein-creatinine ratio; PRA = panel reactive antibodies; RRT = renal replacement therapy; SD = standard deviation; UrSt = ureteral stent.

a Lupus nephritis, infection, reflux nephropathy, etc.

### 3.2 Primary and secondary endpoints

Overall, 49.3% (*n* = 216) of patients received a prophylactic UrSt during KTX surgery, whereas 50.7% (*n* = 222) did not. BKPyV-DNAemia–positive patients had slightly higher rates of UrSt implantation (51.6%) than BKPyV-DNAemia–negative patients (47.0%, *p* = 0.34). UrSt indwelling time was significantly longer in BKPyV-DNAemia–positive patients (*p* < 0.01; Table 1), while the time to first DNAemia did not vary by UrSt implantation (hazard ratio [HR] 1.14, 95% confidence interval [CI] 0.87–1.49, *p* = 0.30).

**Fig. 1:**
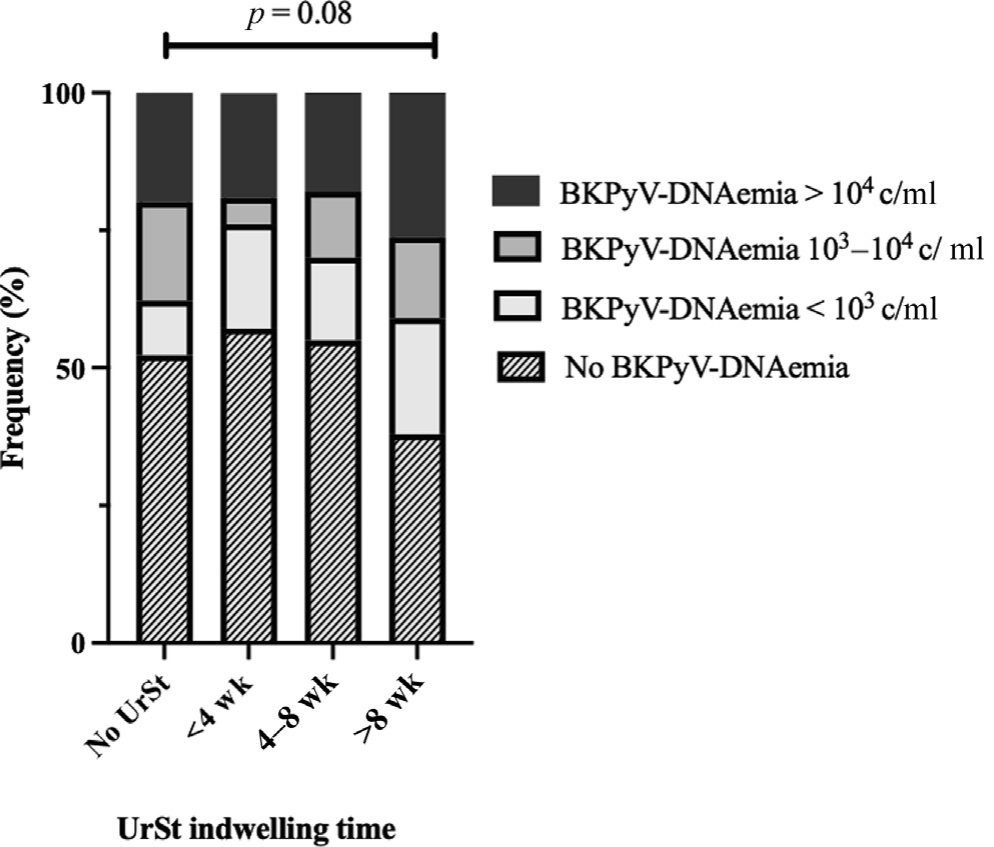
BK polyomavirus (BKPyV)-DNAemia levels and ureteral stent (UrSt) indwelling time. Patients with UrSt indwelling time beyond 8 wk exhibited an elevated rate of high‐level BKPyV‐DNAemia (*p* = 0.06). c/ml = copies per milliliter.

The detection of even small concentrations of BKPyV-DNA (at least 10^2^ c/ml) was highest in the group with UrSt indwelling times of over 8 wk (62%, *p* = 0.06; Table 2). High-level BKPyV-DNAemia (>10^4^ c/ml), a recognized marker of the biopsy-proven BKPyVAN, appeared more frequently among patients with prolonged UrSt indwelling times. Specifically, the highest rate was observed in those with stents in place for >8 wk (26.3%), compared with 19.8% in patients without a stent, 21% in those with stents removed within 4 wk, and 18% in the 4–8-wk group. Although the difference did not reach statistical significance (*p* = 0.08), this pattern suggests a possible association between a longer stent duration and an increased risk of significant viremia (Fig. 1). Among cases with available DNAuria data (∼25% of cohort), BKPyV-DNAuria correlated strongly with DNAemia 1 mo later (rho 0.55–0.83, *p* < 0.001). The incidence of biopsy‐proven BKPyVAN was highest in patients with a long duration of an indwelling UrSt (*p* = 0.49), despite the low event rate (Table 2).

**Table 2:** Comparison of key primary and secondary BKPyV outcomes across the duration of UrSt indwelling categories

Outcomes	No UrSt (*n* = 222)	UrSt <4 wk (*n* = 21)	UrSt 4–8 wk (*n* = 100)	UrSt >8 wk (*n* = 95)	*p* value
BKPyV-DNAemia (>10^2^ c/ml), *n* (%)	106 (47.7)	9 (42.9)	45 (45.0)	59 (62.1)	0.06
High‐level BKPyV‐DNAemia (>10^4^ c/ml), *n* (%)	44 (19.8)	4 (21)	18 (18)	25 (26.3)	0.08
First BKPyV-DNAemia (mo), median (IQR)	3.0 (2.0–6.0)	6.0 (3.0–9.0)	3.0 (2.0–4.0)	3.0 (2.0–5.0)	0.26
Peak BKPyV-DNAemia (c/ml), median (IQR)	6.35 × 10^3^ (1.20 × 10^3^–3.53 × 10^4^)	1.80 × 10^3^ (5.20 × 10^2^–4.90 × 10^4^)	3.30 × 10^3^ (4.55 × 10^2^–5.50 × 10^4^)	6.50 × 10^3^ (2.30 × 10^2^–4.70 × 10^4^)	0.85
Biopsy-proven BKPyVAN, *n* (%)	11 (4.9)	1 (4.7)	7 (7.0)	9 (9.5)	0.49

BKPyV = BK polyomavirus; BKPyVAN = BKPyV‐associated nephropathy; BKPYyV-DNAemia = detection of BKPyV DNA in plasma; IQR = interquartile range; UrSt = ureteral stent.

High BKPyV-DNAemia denotes patients with peak viral loads *of* >10^4^ copies/ml (c/ml). Peak BKPyV‐DNAemia is the maximum plasma viral load (c/ml) recorded.

### 3.3 UrSt indwelling time is a risk factor for BKPyV-DNAemia

**Fig. 2:**
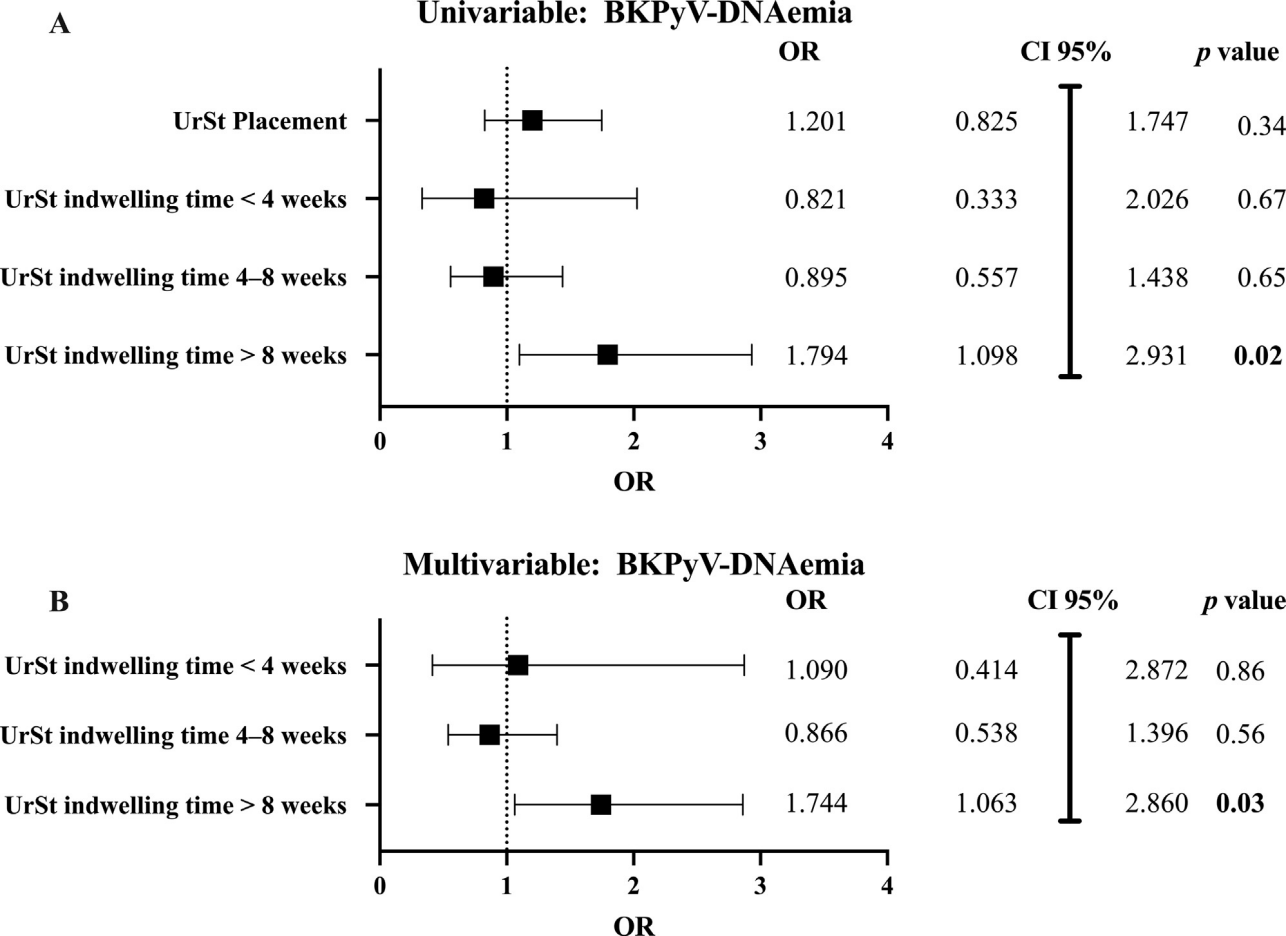
Risk factor analysis for BK polyomavirus (BKPyV)-DNAemia. (A) In a univariable analysis, a long ureteral stent (UrSt) indwelling time (>8 wk) was a statistically significant predictor of BKPyV-DNAemia (*p* = 0.02). (B) A long UrSt indwelling time remained significant in a multivariable analysis, adjusted for the effects of age at KTX, sex, use of tacrolimus, and use of glucocorticoids (*p* = 0.03). BKPyVAN: BK polyomavirus–associated nephropathy; CI = confidence interval; KTX = kidney transplantation; OR = odds ratio.

As shown previously, prolonged UrSt indwelling times in KTX patients were significantly associated with BKPyV-DNAemia (*p* < 0.01). To further assess the impact of UrSt implantation and UrSt indwelling time on BKPyV-DNAemia, a binary logistic regression model was applied. While overall UrSt placement was not significantly associated with any BKPyV-DNAemia (univariable odds ratio [OR] 1.20, 95% CI 0.82–1.75, *p* = 0.34), there was a dose-dependent relationship between indwelling times and BKPyV-DNAemia (*r* = 0.19, *p* < 0.01). In a univariable analysis, short and intermediate UrSt indwelling times showed no association (OR 0.82, 95% CI 0.33–2.07, and OR 0.89, 95% CI 0.56–1.44, respectively), whereas prolonged indwelling time (>8 wk) showed a 1.79-fold increase in the odds of BKPyV-DNAemia (OR 1.79, 95% CI 1.09–2.93, *p* = 0.02). This remained statistically significant even after adjustment for the effects of sex, age at KTX, tacrolimus, and glucocorticoid use (adjusted OR 1.74, 95% CI 1.06–2.86, *p* = 0.03; Fig. 2, Fig. 2, and Supplementary Fig. 2). Owing to low event rates of biopsy-proven BKPyVAN, a regression analysis for this endpoint was not performed.

### 3.4 Association between prolonged UrSt indwelling time (>8 wk) and BKPyV-DNAemia and BKPyVAN

**Fig. 3:**
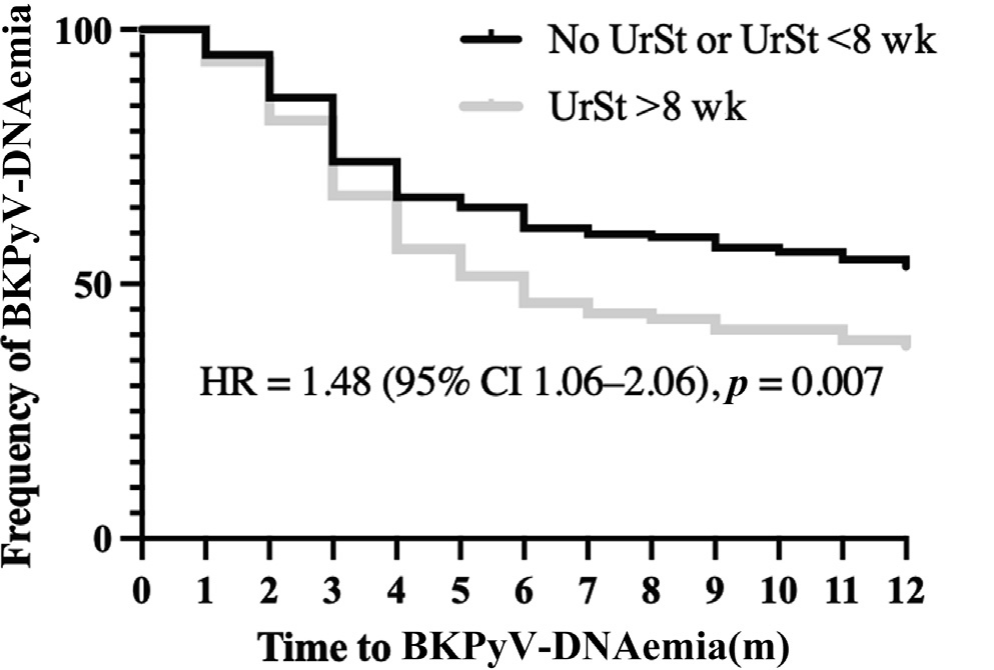
Kaplan-Meier curve for time to BK polyomavirus (BKPyV) infection stratified by ureteral stent (UrSt) indwelling time (>8 vs ≤8 wk, or none). The figure shows the proportion of patients remaining free of BKPyV-DNAemia over 12 mo of follow-up. CI = confidence interval; HR = hazard ratio.

We stratified patients by UrSt indwelling time into two groups: no UrSt or short/intermediate UrSt indwelling time (≤8 wk) versus long UrSt indwelling time (>8 wk). When comparing these groups, the proportion of patients with BKPyV infection was significantly higher in the group with UrSt indwelling time of >8 wk (62.1% vs 46.6%, *p* = 0.01). Biopsy-proven BKPyVAN occurred in nine of 95 (9.5%) patients with prolonged UrSt indwelling time versus 19 of 343 (5.5%) with short indwelling time or no UrSt implantation (*p* = 0.17). The Kaplan-Meier curve demonstrated a significantly shorter BKPyV infection‐free interval in patients with UrSt indwelling time of >8 wk (HR 1.48, 95% CI 1.06–2.06, *p* = 0.007; Fig. 3).

### 3.5 Subgroup analysis (pre- vs post-2017 transplant era)

Of the 438 recipients, 252 (57.5%) underwent KTX before 2017 and 186 (42.5%) after 2017. BKPyV-DNAemia rates were slightly higher in the pre-2017 cohort (55.7% vs 44.3%; *p* = 0.44), whereas UrSt utilization increased markedly after 2017 (94.6% vs 15.9%; *p* < 0.001; Supplementary Fig. 3). The rates of BKPyV infection in the pre-2017 cohort were similar among stented (52.5%) and unstented (47.6%) patients (*p* = 0.57). The interaction model confirmed that prolonged UrSt indwelling (>8 wk) remained an independent predictor of BKPyV‐DNAemia (*B* = 0.77, *p* = 0.01), corresponding to a 2.17‐fold increase in odds (95% CI 1.18–3.98). Neither the transplant era indicator (after 2017) nor its interaction with UrSt indwelling time reached significance (interaction term *B* = –0.54, OR 0.58, 95% CI 0.19–1.75, *p* = 0.34), indicating that the effect of extended indwelling time on DNAemia did not differ before versus after the 2017 guideline change. All other covariates—including sex, age at KTX, and use of tacrolimus or glucocorticoids—were nonsignificant in this model (all *p* > 0.1).

## 4 Discussion

Ureteral stenting is used routinely in KTX patients to reduce postoperative complications. However, emerging data suggest that it may increase the risk and severity of BKPyVAN [37,38]. In our matched retrospective study of 438 KTX recipients, overall UrSt placement was not associated with BKPyV-DNAemia or BKPyVAN. However, prolonged UrSt indwelling (>8 wk) was a significant predictor of BKPyV-DNAemia in both univariable (OR 1.79) and multivariable (adjusted OR 1.74) models. A time-to-event analysis showed that UrSt indwelling time of >8 wk shortened the infection-free interval (HR 1.48, 95% CI 1.06–2.06).

These findings differ from those of earlier studies, often limited by small cohorts and heterogeneous UrSt use. Wingate et al. [25] reported increased BKPyV-DNAemia for UrSt indwelling time of >3 wk in an unmatched cohort, while a multicenter analysis found an adjusted HR of 1.36 despite only 14.9% having BKPyV-DNAemia [26]. Wingate et al. [25] did not stratify indwelling time further; however, it is plausible that their “>3 wk” category encompassed a heterogeneous mix of patients—some with moderate (4–8 wk) and others with prolonged (>8 wk) indwelling times, suggesting that the risk may be concentrated in longer durations. Other studies suggested links between a UrSt and BKPyV replication, but were limited by binary analyses or missing duration data. Siparsky et al. [24] found a 3.2-fold increase in DNAemia with the removal of the UrSt at 4 wk, but binary grouping and mixed regimens may have masked effects. Hashim et al. [27] reported a modest rise (22% vs 16%) without duration data. Maliakkal et al. [26] found an increased risk (median removal ∼42 d), while Thomas et al. [29] and Kayler et al. [35] also reported associations without timing info. On the contrary, one multicenter study of KTX recipients with hepatitis C virus–positive donors found no link between a UrSt and BKPyV-DNAemia (HR 1.10, *p* = 0.69), in line with our results [39]. Additionally, Eder et al’s. [40] meta-analysis—the largest to date—also showed no association between UrSt implantation and BKPyVAN-DNAemia.

Regarding biopsy-proven BKPyVAN, while we observed higher rates in UrSt patients, our study was not powered to detect statistically significant differences between stented and unstented patients. One single-center study from 2007—comparing 20 cases of BKPyVAN with 46 controls—showed that ureteric stenting increased the risk of BKPyVAN [29], while Demey et al. [20] did not identify a UrSt as a risk factor. Since BKPyVAN follows sustained DNAemia, an indirect link is plausible but unconfirmed.

After the EAU’s 2017 recommendation for routine use of a UrSt, stent use rose from 15.9% to 94.6%; yet, BKPyV infection and BKPyVAN rates remained similar. Furthermore, the BKPyV risk and stent effect remained consistent across transplant eras. After 2017, 15 of 16 BKPyVAN cases had a UrSt, while most pre-2017 cases did not. These findings argue against an increased risk from routine UrSt use if removed within 8 wk.

The strengths of our study include our large matched sample, high DNAemia rate, and uniform IS. By stratifying UrSt indwelling times into three discrete intervals, we were able to pinpoint the narrow window in which prolonged UrSt placement influences the risk of BKPyV-DNAemia modestly, and our comprehensive multivariable models adjusting for key covariates (sex, age at KTX, and tacrolimus and glucocorticoid use) bolstered the validity of our findings. Finally, a pre- versus post-2017 subgroup analysis—bracketing the EAU’s guideline change—confirmed that the marked increase in stenting did not translate into higher rates of BKPyV infection or BKPyVAN. Limitations include the retrospective design, potential unmeasured confounders, and possible selection bias during matching, including baseline differences in IS, which were adjusted for in the multivariable analysis. We also acknowledge not assessing the donor/recipient origin of the BKPyV infection as a limitation of our study.

## 5 Conclusions

In summary, UrSt indwelling time of >8 wk was associated with an increased risk of BKPyV-DNAemia and earlier reactivation of BKPyV after KTX. While overall UrSt placement was not associated with BKPyV-DNAemia or BKPyVAN, our analysis highlights the importance of removing the UrSt within 8 wk after KTX to reduce this risk.

  ***Author contributions*:** Željko Kikić had full access to all the data in the study and takes responsibility for the integrity of the data and the accuracy of the data analysis.

  *Study concept and design*: Omić, Hoffmann, Kikić.

*Acquisition of data*: Omić, Hoffmann.

*Analysis and interpretation of data*: Omić, Hoffmann, Kikić, Shariat.

*Drafting of the manuscript*: Omić, Hoffmann, Eder, Kikić, Shariat.

*Critical revision of the manuscript for important intellectual content*: Kikić, Gerges, Shariat.

*Statistical analysis*: Omić, Hoffmann, Kikić.

*Obtaining funding*: None.

*Administrative, technical, or material support*: Strassl, Shariat.

*Supervision*: Kikić, Shariat.

*Other*: None.

  ***Financial disclosures:*** Željko Kikić certifies that all conflicts of interest, including specific financial interests and relationships and affiliations relevant to the subject matter or materials discussed in the manuscript (eg, employment/affiliation, grants or funding, consultancies, honoraria, stock ownership or options, expert testimony, royalties, or patents filed, received, or pending), are the following: None.

  ***Funding/Support and role of the sponsor*:** None.
