# Supplementary material for: Prolonged Prophylactic Ureteral Stent Placement and BK Polyomavirus Infection After Renal Transplantation—A Retrospective Case-control Study
Source: Eur Urol Open Sci. 2025 Nov 4;82:121–7. doi: 10.1016/j.euros.2025.10.014 (PMC12637267; doi:10.1016/j.euros.2025.10.014)
Supplement: Supplementary Data 1 [file mmc1.docx]

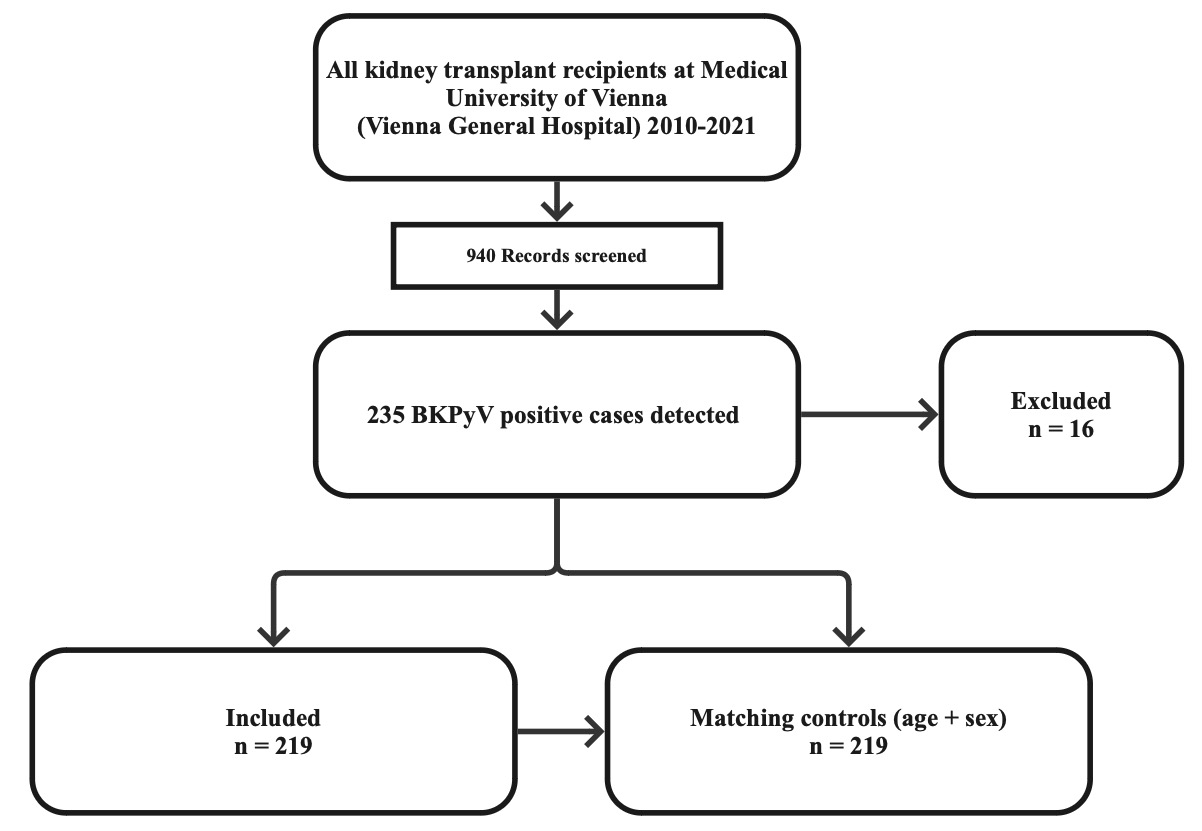


**Supplementary Figure 1. Study Flowchart.** Sixteen patients were excluded due to missing parameters and/or lost to follow-up. BKPyV: BK Polyomavirus

**
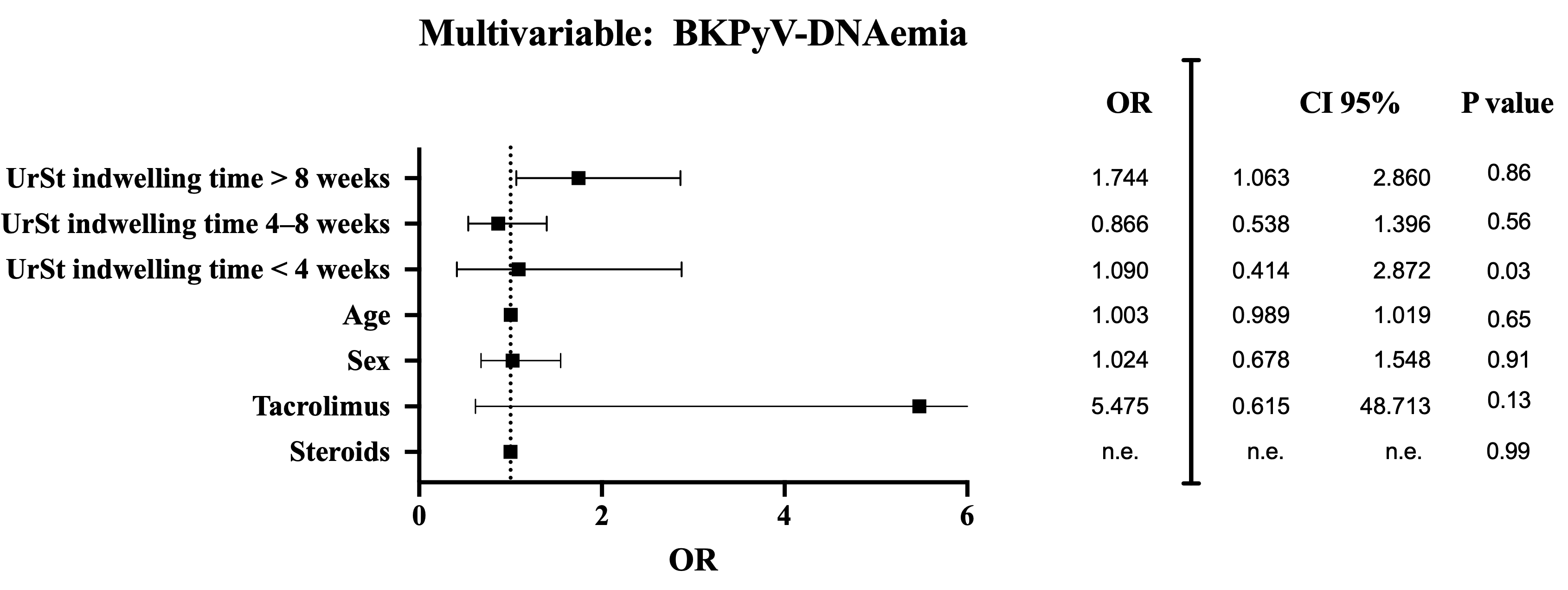
**

**Supplementary Figure 2**. **Multivariable logistic regression analysis of risk factors for BK polyomavirus DNAemia**. Odds ratios (OR) and 95% confidence intervals (CI) are shown for ureteral stent (UrSt) indwelling time and covariates. UrSt >8 weeks was independently associated with BKPyV-DNAemia (aOR 1.74; 95% CI 1.06–2.86). Glucocorticoid use was nearly universal (>98%); logistic regression yielded an unstable estimate with inflated OR and wide CI due to quasi-complete separation, and is therefore reported as n.e. (not estimable).

**Supplementary Figure 3.** **Comparison of BK Polyomavirus (BKPyV) outcomes and ureteral stent (UrSt) use before and after the 2017 European Association of Urology (EAU) recommendation of routine UrSt placement in kidney transplantation (KTX).** Bar charts show the proportion of KTX recipients in the pre-2017 (n = 252) and post-2017 (n =186) cohorts with BKPyV-DNAemia, BKPyV-associated nephropathy (BKPyVAN), and UrSt placement. Data are presented as percentages. BKPyV infection rates did not differ significantly between cohorts (55.7% vs. 44.3%, p=0.44). UrSt placement increased markedly after 2017 (15.9% vs. 94.6%, p<0.001). BKPyVAN incidence was higher but not significant in post-2017 era (4.8% vs. 8.6%, p=0.10). BKPyV: BK Polyomavirus, BKPyVAN: BK Polyomavirus associated nephropathy, KTX, kidney transplantation, ns: not significant, UrSt: ureteral stent.
